# Surface-Modified Nanocarriers Encapsulating Brucine and Nigella Sativa Oil: A Novel Approach to Solid Tumor Therapy

**DOI:** 10.3390/ph18101495

**Published:** 2025-10-04

**Authors:** Heba S. Elsewedy, Tamer M. Shehata

**Affiliations:** 1 Department of Pharmaceutical Sciences, College of Pharmacy, AlMaarefa University, Diriyah 13713, Saudi Arabia; 2 Research Center, Deanship of Scientific Research and Post-Graduate Studies, AlMaarefa University, Diriyah 13713, Saudi Arabia; 3 Department of Pharmaceutical Sciences, College of Clinical Pharmacy, King Faisal University, Hofuf 31982, Saudi Arabia

**Keywords:** nanocarrier, PEGylation, nanoemulsion, drug targeting, anti-tumor activity

## Abstract

**Background:** Using natural substances for cancer therapy has attracted considerable interest due to their safety and reduced systemic toxicity. Nigella sativa (NS) oil, a traditional natural oil rich in bioactive compounds, possesses significant therapeutic potential. Brucine (BR), an alkaloid, exhibits potent cytotoxicity against various cancer cell lines; however, its poor selectivity and high systemic toxicity limit its clinical application. **Objective:** To overcome these challenges, this study aimed to enhance drug delivery and improve therapeutic efficacy. **Method:** A PEGylated nanoemulsion (NE) incorporating NS and BR was developed and characterized for particle size, size distribution, zeta potential, viscosity, and drug content. The in vitro release of BR was evaluated both with and without serum incubation. A quantitative amount of serum protein associated with the surface of the NE was estimated, and a hemolytic safety assay was carried out. Finally, an in vitro cytotoxicity study was conducted, and the in vivo anti-tumor effect of the developed PEGylated BR-loaded NE was evaluated and compared with its naked counterpart. **Result:** The developed PEGylated BR-loaded NE possessed favorable characteristics as a nanocarrier for parenteral administration, with a particle size of 188.5 nm, a zeta potential of −1.61, a viscosity of 3.4 cP, and 99% drug content uniformity. It released up to 60.4% of BR over 12 h, while only 18.4 µg/µmol of the total lipids were adsorbed on the surface of the formulation, compared with 54.5 µg/µmol for the naked counterpart. The PEGylated NE was safe, inducing less than 5% of hemolysis, and displayed substantial inhibition of MDA cell growth. **Conclusions:** The PEGylated NE achieved a significant reduction in tumor volume, suggesting that PEGylated NE may serve as a promising platform for enhancing anti-tumor activity.

## 1. Introduction

Cancer is a group of diseases characterized by irregular proliferation and the spread of abnormal cells throughout the body [1]. It can affect any tissue or organ, leading to serious complications, and is, therefore, regarded as one of the most life-threatening diseases [2]. The etiology of cancer is complicated, encompassing a variety of causes, mainly including genetic susceptibilities, environmental influences, and lifestyle factors like nutrition, tobacco use, and physical exercise [3,4]. Various complications may result from cancer, which can arise from the illness itself or from its treatment. Consequently, finding a strategy that manages the disease while controlling its drawbacks is crucial. Advances in research and treatment modalities, including surgery, radiation therapy, immunotherapy, chemotherapy, and targeted therapies, have markedly enhanced patient outcomes [5]. Nevertheless, several challenges remain, especially regarding the management of treatment-related adverse effects.

Nanotechnology is an approach that is applied extensively in the medical field. It concerns the manufacturing and utilization of systems at a nanoscale [6]. It demonstrates great influence in improving the effectiveness of medications and lowering their side effects. Regarding this concern, drugs can be specifically targeted to tumor cells using nanocarriers, leaving healthy tissues protected [7]. Drug targeting is a strategy used to distribute therapeutic medication more efficiently to a particular site, reducing side effects and enhancing effectiveness [8]. Two primary pathways of drug delivery are addressed: active and passive targeting. The mechanism of active targeting involves the conjugation of affinity ligands to the surface of nanocarriers to enhance their retention at the target site [9]. On the other hand, passive targeting denotes the accumulation of drugs in particular tissues or organs and is influenced by many physiological and biochemical processes, in contrast to active targeting [10]. Drug targeting offers an efficient strategy for drug delivery by enabling selective accumulation in specific regions, reducing side effects, prolonging the half-life of the nanocarriers, and improving bioavailability [11]. As broadly investigated, passive targeting depends on a natural phenomenon known as the enhanced permeability and retention (EPR) effect, which is characteristic for most solid tumors [12]. This phenomenon prolongs the circulation time of the drug-loaded nanocarrier, which could increase its chances of reaching the tumor [13]. The major limitation of intravenous injection is that the reticuloendothelial system (RES) quickly removes the nanocarrier systems from blood circulation. One potential method for improving the blood residence time of nanocarriers is to modify their surfaces using hydrophilic polymers like PEG and PVA [14,15]. Coating the nanocarriers with a hydrophilic polymer can prevent serum opsonin from activating RES.

A number of nanocarriers have been developed as delivery systems for delivering drugs to their targeted sites, including liposomes, niosomes, nanoparticles, nanoemulsions (NEs), and others [16]. NE is a surfactant-stabilized colloidal dispersion formed from two immiscible liquids, usually water and oil [17]. NEs have attracted a lot of interest due to their unique properties and advantages in improving drug delivery [18]. Several studies were investigating the delivery of several chemotherapeutic agents for treating cancer using NE as a nanocarrier [19,20]. Brucine (BR) is one of the substances that attracted attention since it exhibited great anticancer activity against cancer cells [21,22,23]. BR has previously been formulated as a nanoparticle, nanoemulsion, niosome, and ethosome for delivery systems, all of which have shown good anticancer activity [24,25,26,27]. It is a natural alkaloid produced from the Strychnos nux-vomica plant and exists in the form of white, crystalline, water insoluble powder [28]. However, the poor solubility of BR (1 g in 1350 mL distilled water) is a great hurdle in its manufacturing [25,29].

Combination therapy involves the utilization of two or more therapeutic agents to effectively address a disorder and enhance the treatment efficacy compared with monotherapy [30]. The utilization of natural products, such as essential oils, especially when integrated with novel drug delivery systems like nanocarriers, is an emerging area of research in oncology [31]. Nigella sativa (NS) or black seed has been widely investigated for anticancer activity owing to its major component of thymoquinone [32], in addition to other bioactive components such as alkaloids, saponins, and polyphenols that enhance its therapeutic efficacy. It was documented that NS oil had the ability to prevent colon carcinogenesis without any adverse reactions [33]. Additionally, studies have shown that NS extract can successfully deactivate breast cancer cells and may play a role in inhibiting skin carcinogenesis. Other investigations demonstrated the anti-proliferative effect of NS constituents against leukemia [34]. It could suppress the development of solid tumors and delay the mortality of the examined animals as reported by Mbarek et al. [35]. Thymoquinone, a major bioactive compound in NS oil, has shown anticancer activity through multiple pathways, one of which involves the inhibition of angiogenesis [36]. Additionally, it could induce apoptosis through the mitochondrial pathway, leading to mitochondrial dysfunction [37]. Other mechanisms include inducing oxidative stress and cell death [38].

In this context, the combination of BR with surface-modified NE prepared using other therapeutic substances such as NS oil has been studied. Previous investigations have explored PEGylated brucine nanoemulsion and Nigella sativa oil separately. To the best of our knowledge, no studies have yet combined BR with NS oil within a single PEGylated nanoemulsion platform. The novelty of the current study lies in the synergistic action of both BR and NS oil, in addition to the strategy of developing the formulation, which is a combination between brucine, Nigella sativa oil, and PEGylation in a single nanoemulsion system. All these points have not been documented previously. The developed PEGylated BR-loaded NE was evaluated for its physicochemical properties. The amount of serum protein adsorbed on the surface of the prepared NE was quantitatively estimated and the formulation was subsequently evaluated for its enhanced anti-tumor efficacy.

## 2. Results and Discussion

### 2.1. Characterization of the Developed BR-Loaded NE

The developed BR-loaded NE preparations were homogeneous, stable, and showed no signs of phase separation when kept either in the refrigerator or at room temperature. Several evaluations were performed including evaluations of particle size, PDI, viscosity, and drug content.

#### 2.1.1. Assessment of Particle Size, Size Distribution and Zeta Potential

**Figure 1:**
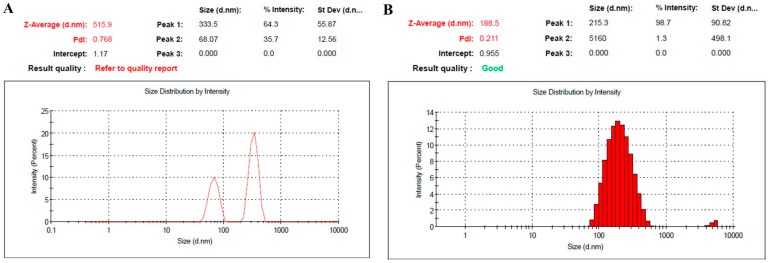
Particle size and PDI of (**A**) naked BR-loaded NE, namely NE1; and (**B**) PEGylated BR-loaded NE, namely NE2.

The results obtained after determining the NE particle size and PDI are displayed in Table 1 and Figure 1. Regarding the particle size, it was assessed to be 516.9 ± 5.2 nm and 188.5 ± 3.3 nm for NE1 and NE2, respectively. Concerning the polydispersability index (PDI), it was found to be 0.768 for NE1 and 0.211 for NE2. It is well known that particle size and PDI are crucial parameters for formulations intended for intravenous administration [39]. Moreover, it was documented that the ideal PDI value that indicates the homogeneity of the particle size in a formulation could be lower than 0.7 [40]. Referring to the results obtained, it was noticed that the particle size and PDI of NE1 were relatively high, revealing heterogeneity of the particle size and a broad size distribution. In contrast, the particle size distribution falls within a narrow range of sizes in the case of NE2, which is considered a positive indicator of formulation stability. These results emphasized the impact of using PEG on the particle size and size distribution of the NE formulation. A medication formulation’s therapeutic efficacy may be impacted by uneven particle sizes. Our findings are in agreement with Jelena et al., who prepared injectable PEGylated nanoemulsion containing curcumin with particle sizes ranging from 106 to 171 nm with a PDI less than 0.15 [41]. Regarding the surface charge of the formulation, the zeta potential was estimated for both NEs. It was noted that there was a remarkable reduction in the negative surface charge of the PEGylated NE compared with its naked counterpart. This could be attributed to the layer of PEG shielding the NE preparation [10,42].

#### 2.1.2. Assessment of Viscosity

Maintaining viscosity within an acceptable range is essential for formulations intended for intravenous administration [43], whereas higher viscosity might cause pain and results in embolism during administration. As shown in Table 1, the viscosity of the developed NE1 and NE2 was 2.8 ± 0.3 and 3.4 ± 0.45 cP, respectively. According to previous studies, the suitable value for the viscosity of the NE was below 3.9 cP [39]. Séguy et al. developed a non-hemolytic nanoemulsion for intravenous administration showing a viscosity of 0.88 cP [44]. Moreover, Aripiprazole nanoemulsion developed for parenteral delivery application showed a viscosity of 3.72 cP [45].

#### 2.1.3. Assessment of Drug Content

As displayed in Table 1, the developed NEs were evaluated for drug content in order to assess the amount of active ingredients in the formulation. The data revealed that the drug content was 98.6 ± 0.7% and 99 ± 1.0% for NE1 and NE 2, respectively, suggesting a uniform distribution of BR throughout all produced NE formulations.

### 2.2. TEM Analysis

**Figure 2:**
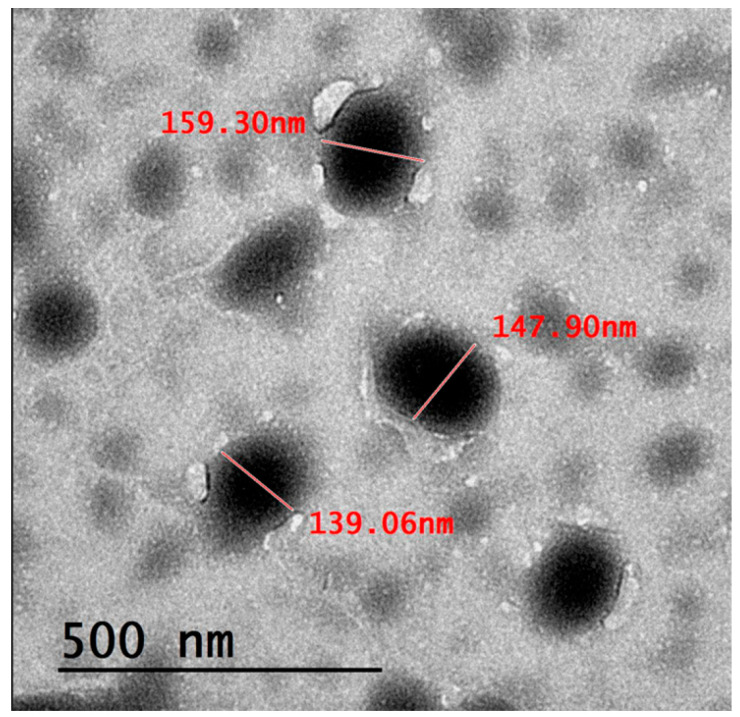
TEM image of the developed PEGylated NE formula.

The image obtained from TEM analysis belonged to PEGylated NE (NE2) and is shown in Figure 2. The TEM Micrograph revealed that the NE droplets were predominately spherical in shape with smooth surfaces, indicating uniform and homogeneous preparation. Further, the particle size detected by TEM analysis appeared to be closely related to the particle size obtained from dynamic light scattering using Malvern zetasizer. Additionally, no aggregation or coalescence was exhibited between droplets, emphasizing the stability of the formulation [46].

### 2.3. Studying In Vitro Release of BR from the Developed NE

**Figure 3:**
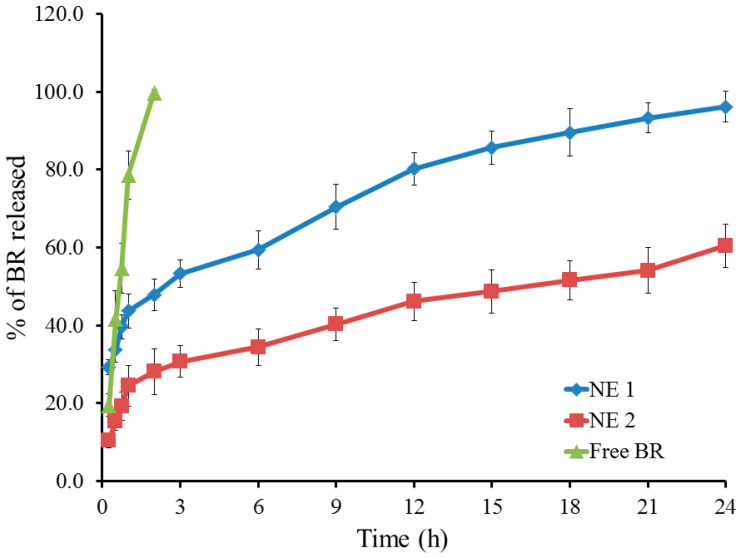
Profile for in vitro release study of BR from NE1 and NE2 compared with free BR for 24 h at 37 °C in PBS pH 7.4. Results are expressed as mean, with the bar showing the S.D. of three experiments.

Figure 3 depicts the in vitro drug release profile of BR from the NE formulations. The amount of BR released from the PEGylated NE (NE2) was 60.4 ± 5.6 following 24 h, while the release from its naked counterpart (NE1) showed 96.2 ± 4.0%. It is evident that the amount of drug released from NE1 was greater than that from NE2, which may be attributed to the lower stability of the naked NE compared with the PEGylated formulation. In addition, the rigidization of the membrane at the PEGylated NE surface may lead to a decrease in the percentage of drug release [47,48].

### 2.4. Studying In Vitro Release of BR from the Developed NE Following Serum Incubation

**Figure 4:**
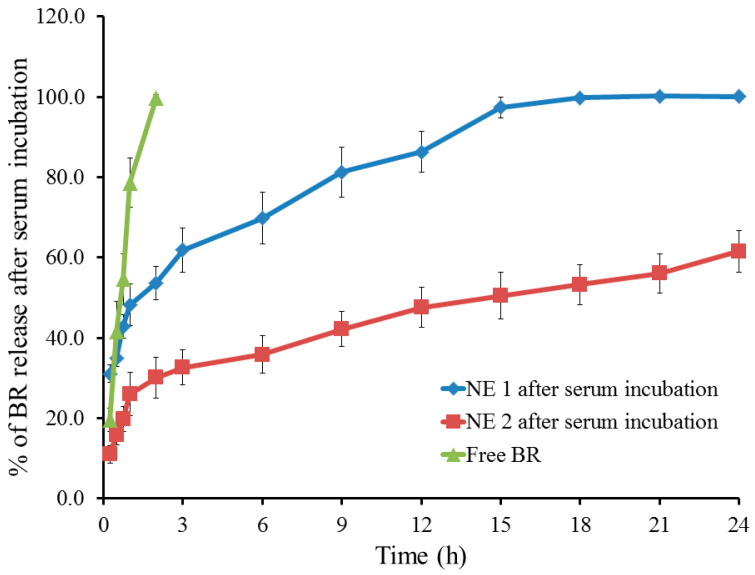
Profile for in vitro release study of BR from NE1 and NE2 compared with free BR for 24 h at 37 °C, following incubation with 10% serum in PBS pH 7.4. Results are expressed as mean, with the bar showing the S.D.s of three experiments.

As depicted in Figure 4, the in vitro release of BR from the developed NE formulations was evaluated after being incubated with an equal volume of 10% serum. As per the data obtained, the amount of BR released from NE1 upon serum incubation reached 97.3 ± 2.6% after 15 h and almost 100% after 18 h; however, in the case of NE2, the amount of BR released was 61.5% following 24 h. It is obvious that the release of the drug from the naked formulation following serum incubation was sped up since it was enhanced at an earlier time. This may be ascribed to the assembly of the membrane attack complex (MAC), which contributes to the development of circular lytic holes and leaky patches on the target surface or the membrane of the foreign pathogen [49]. This plausible explanation was documented previously by Ellsworth et al., who confirmed that lytic poreswere formed on the cell as a result of MAC assembly [50]. However, this explanation remains speculative, since no experimental validation was performed regarding MAC formation, in the current study. Moreover, it was earlier stated that serum protein could attack the nanocarriers leading to disruption of the formulation, increasing the release of the drug from the naked formula compared with its PEGylated compartment. This can be explained by the significant adsorption of complement component C3 and immunoglobulin G (IgG) onto the surface of the naked niosome [51].

### 2.5. Quantitative Determination of Serum Protein Adsorbed onto the Surface of NE

**Figure 5:**
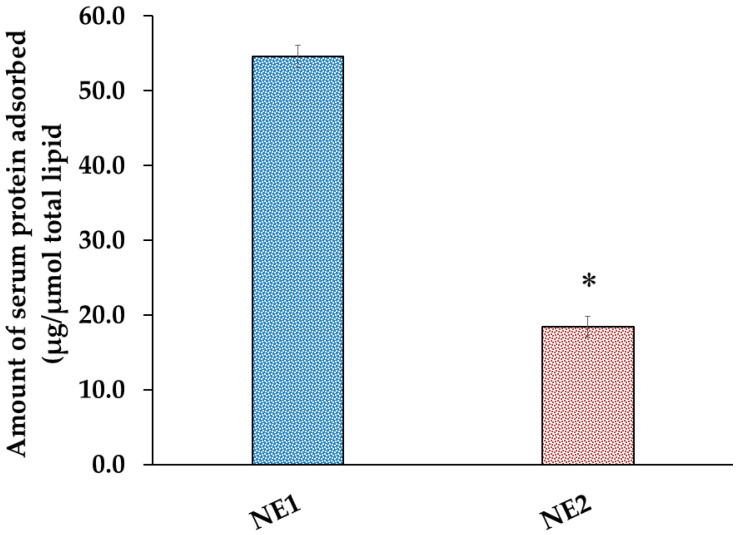
Profile of the total amount of serum proteins adsorbed on the surface of PEGylated NE (NE2) and its naked counterpart (NE1). Results are displayed as the mean, with the bar showing S.D.s of three trials. * *p* < 0.05, compared with NE2.

To assess the amount of serum protein that might bind or adsorb onto the surface of the nanocarriers, a quantitative determination was carried out. This study is essential for understanding the interactions between NEs and biological molecules [52]. It was noted from Figure 5 that the amount of serum protein associated with the surface of NE2 was (18.4 ± 1.3 µg/µmol total lipid) in contrast to its naked counterpart NE1 that exhibited a surface adsorbed quantity (54.5 ± 1.4 µg/µmol total lipid). It is evident that the overall amount of serum proteins bound to the surface of the PEGylated formulation was substantially less than that bound to its naked counterpart. In fact, the presence of PEG on the surface of NE plays a vital role in shielding the surface of the NE, making it unrecognizable to the serum opsonin and consequently, reducing the amount of serum protein attached to the surface of the nanocarrier [42]. This phenomenon, known as fixed aqueous layer thickness (FALT), is caused by PEGylation [53].

### 2.6. Hemolytic Activity

**Figure 6:**
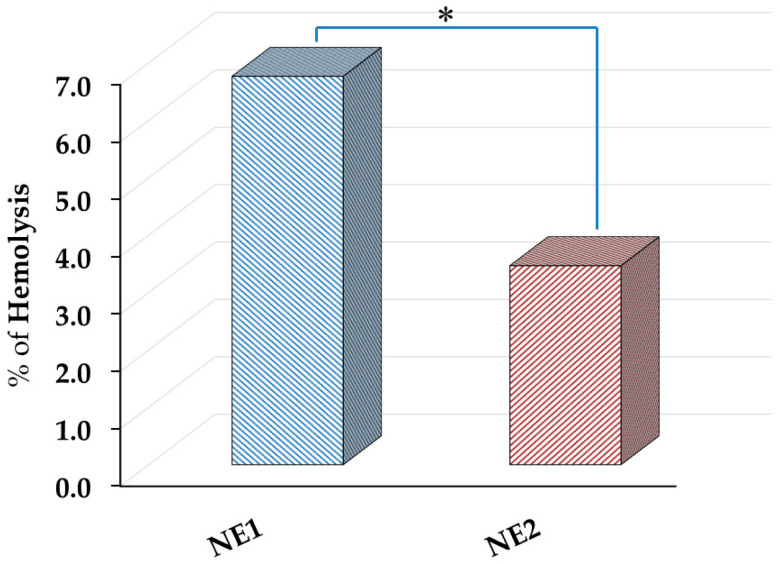
Hemolytic activity of BR-loaded NE preparations after incubation with rat red blood cells (2% *w*/*v* in PBS) at 37 °C for 30 min. Results are stated as the mean ± S.D. of three experiments. * *p* < 0.05, compared with naked counterpart (NE1). All data were compared with Triton-X 100, which provided 100% hemolysis.

The study of hemolytic activity is a critical assay, especially for the evaluation of drugs intended for intravenous administration. This test is commonly performed to assess the safety and compatibility of the preparation, specifically to determine whether it causes the lysis of red blood cells and subsequent release of hemoglobin [54]. Accordingly, the hemolytic activity test was performed and the data is shown in Figure 6. Following 30 min of incubation, both NE1 and NE2 exhibited minimum hemolysis of less than 5%, which indicates that these formulations are non-hemolytic, suitable for intravenous administration, and sufficiently safe [55].

Since the PEGylated NE formulation (NE2) showed a minimal percentage of drug release and low serum protein adsorption, it was suggested to assess its pharmacological anti-tumor influence.

### 2.7. In Vitro Cytotoxicity Study: MTT Assay

**Figure 7:**
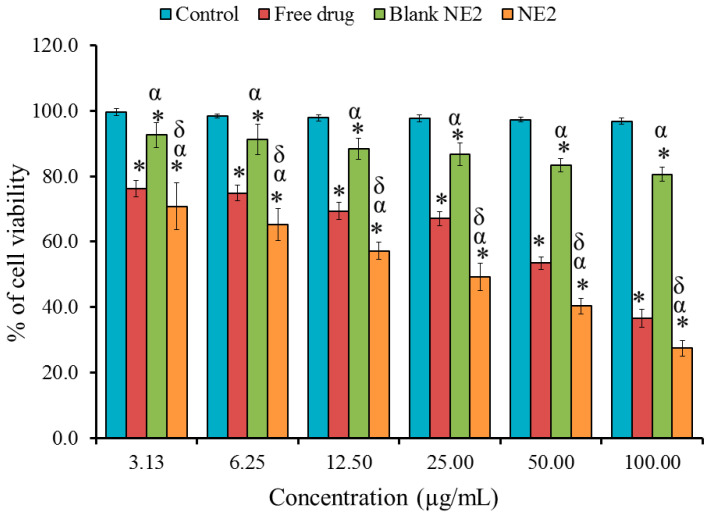
Profile showing percentage of cell viability for control, free BR, blank NE2, and NE2 against MDA-MB-231 cells incubated for 48 h. Results are stated as the mean ± S.D. * Statistically significant from control (*p* < 0.05). α Statistically significant from free drug (*p* < 0.05). δ Statistically significant from blank NE2 (*p* < 0.05). Results are expressed as the mean ± SD of three experiments, *n* = 3.

In order to evaluate the cell viability of BR-loaded NE against MDA-MB-231 cells, the colorimetric MTT test was exploited. As exhibited in Figure 7, a significant decline was perceived in the growth of the cells treated with NE2, showing an IC50 of 36.14 ± 6.04 µg/mL if compared with cells treated with free BR that possessed an IC50 of 64.57 ± 5.71 µg/mL (*p* < 0.05). This finding proposed that the cytotoxic action of BR is more enhanced when incorporated into the NE compared with its free form. Furthermore, the blank NE2 showed a marked decline in the viability of MDA cells. Remarkably, a significant difference between the control and blank NE was perceived, which may be owed to the composition of NEs. As a result of better water solubility and increased cellular absorption of BR when formulated in NE, BR-NE showed a significant dose-dependent reduction in cancer cell viability [56].

### 2.8. In Vivo Studies

#### Evaluating the Anti-Tumor Activity of BR-Loaded NE

**Figure 8:**
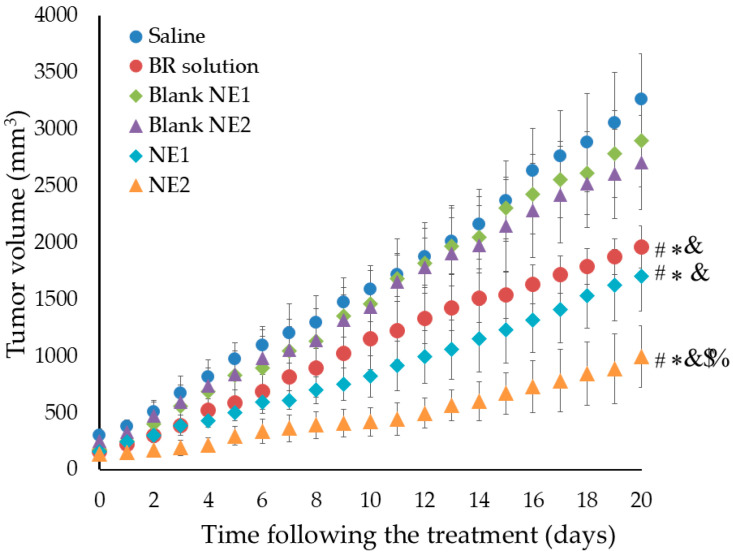
Tumor volume response to BR-loaded-NE in mice bearing MDA solid tumors. # Statistically significant from the saline group (*p* < 0.05). * Statistically significant from the blank NE1 group (*p* < 0.05), and & statistically significant from the blank NE2 group (*p* < 0.05). $ Statistically significant from the BR solution groups (*p* < 0.05). % Statistically significant from the NE1 group (*p* < 0.001). Results are expressed as the mean ± SD of three experiments, *n* = 8.

The anti-tumor efficacy of BR and NS was estimated by measuring the tumor volume in mice bearing MDA solid tumors, following treatment with tested formulations. As depicted in Figure 8, the tumor volume after 20 days was 3265.6, 2897.2, 2703.4, 1961.6, 1706.2, and 997.4 mm^3^, following treatment with saline, blank NE1, blank NE2, BR solution, NE1, and NE2, respectively. Significant tumor growth inhibition was distinguished in the group treated with BR-loaded NE, namely the NE2 formulation, compared with other formulations *p* < 0.001. The observed effect can mostly be attributed to several factors, including the small particle size of the PEGylated NE formulation, which facilitates passive targeting and provides the ability of higher drug accumulation within the tumor cells due to the EPR effect. Furthermore, the PEGylation of NE forms a stealth layer around the nanocarrier that protects it from being recognized and cleared by the phagocytosis process [57]. Consequently, the systemic circulation of the PEGylated NE would be prolonged, providing more residence time in the tumor cells [41]. Moreover, the principal component of NS, thymoquinone, was expected to provide a possible synergistic anti-tumor effect with BR since the reduction in the tumor volume caused by NE2 was significantly higher than the reduction caused by the BR solution. All the previous reasons emphasized the great influence of PEGylation and the anti-tumor effect of NS. However, specific synergy analysis investigations will be required in future studies to confirm this probable synergistic interaction. It is worth noting that the study was performed solely in the MDA-MB-231 breast cancer cell line, with no investigations performed on non-cancerous cells. Future studies are therefore needed to evaluate the safety and selectivity of the formulation.

## 3. Materials and Methods

### 3.1. Materials

BR was acquired from Alpha Chemika, (Mumbai, India). NS oil was supplied from Oud Milano Co., Ltd., Milan, Italy. Egg yolk phosphatidylcholine (EPC), cholesterol, and oleic acid were procured from Sigma Aldrich (St. Louis, MO, USA). Distearoyl phosphatidylethanolamine-N-[methoxy poly (ethylene glycol)-2000] (PEG-DSPE) was acquired from Lipoid LLC, (Newark, NJ, USA). Fetal bovine serum (FBS) and Dulbecco’s modified Eagle’s medium (DMEM) were supplied from Sigma Aldrich (St. Louis, MO, USA). Total protein and Total Lipid Colorimetric kits were bought from United Diagnostics Industry, (Dammam, Saudi Arabia). Tetrazolium dye (MTT reagent) was procured from Loba Chemie (Mumbai, India). All other reagents were of the finest grade available.

### 3.2. Formulation of NE

As shown in Table 2, the required amounts of all ingredients were displayed and used to prepare the BR-loaded NE. Two phases were prepared: an oily phase incorporating NS oil, drug, cholesterol, and oleic acid and an aqueous phase containing EPC. Each phase was mixed well using a classic advanced vortex mixer (VELP Scintifica, Lombardy, Italy) and heated. During homogenization at 15,000 rpm for 15 min using a high shear homogenizer (T 25 digital Ultra-Turrax, IKA, Staufen, Germany), the aqueous phase was gradually added to the oily phase. Instantly, BR-loaded NE was formed and subjected to sonication to attain the desired particle size using a probe sonicator (XL-2000, Qsonica, Newtown, CT, USA) [25]. Two NE formulations were developed, one containing PEG-DSPE to form PEGylated NE (NE2) and the other formulated without PEG-DSPE, referred to as the naked NE (NE1).

### 3.3. Characterization of the Developed BR-Loaded NE

#### 3.3.1. Assessment of Particle Size, Size Distribution, and Zeta Potential

Size distribution and zeta potential are essential parameters in the development and assessment of nanocarriers, particularly in the field of cancer drug delivery [58]. These characteristics substantially affect the stability, effectiveness, bioavailability, and targeting abilities of the nanocarriers. Using a Zetasizer Nano ZS device (Malvern Instruments Ltd., Worcestershire, UK), the particle size, size distribution (PDI), and zeta potential of BR-loaded NEs were established. Dynamic light scattering at 25 °C and a scattering angle of 90°, along with electrophoretic mobility measurements, were used to determine the particle size and zeta potential of the NEs, respectively [59,60]. Briefly, 10 µL of the sample was diluted in 3 mL of distilled water using a disposable cuvette. All the measurements were performed for three measurements of the same batch, and the results are expressed as mean ± standard deviation (SD).

#### 3.3.2. Assessment of Viscosity

Viscosity is a key factor for determining the behavior, stability, and overall performance of the NE system. Accordingly, the viscosity of the formed NE was assessed using a Brookfield viscometer (DV-II+ Pro, Middleboro, MA, USA) with spindle No. 61 at 25 ± 0.3 °C [61]. All the measurements were performed for the same batch in triplicate, and the results have been expressed as mean ± standard deviation (SD).

#### 3.3.3. Assessment of Drug Content

The quantity of the active pharmaceutical ingredient in a formulation is referred to as the drug content. Evaluating the drug content is essential to ensure that the formulation delivers the correct dosage of the drug and retains the desired therapeutic effect [62]. Additionally, it guarantees that the formulation is consistent and efficient for use by patients. To evaluate the drug content, 0.1 mL of the prepared BR-loaded NEs were diluted with phosphate-buffer saline (PBS). Using a UV Spectrophotometer (JENWAY 6305, Bibby Scientific Ltd., Staffs, UK), the drug content was calculated at λmax 264 nm [25]. All the measurements were performed for the same batch in triplicate, and the results have been expressed as mean ± standard deviation (SD).

### 3.4. Transmission Electron Microscopy (TEM Analysis)

TEM analysis was performed to estimate the morphology of the prepared PEGylated NE. In brief, a sample of the formulation was sonicated using an ultrasonicator (Crest Ultrasonics Corp., Ewing Township, NJ, USA) for 10 min. Later, a few drops of the sample were overloaded on a carbon-coated copper grid and left to dry. The grid was then stained with a few drops of phosphotungstic acid (1%) and left to dry. The grid loaded with the prepared sample was examined using HR-TEM (JEOL, JEM-2100, Tokyo, Japan) [63].

### 3.5. Studying In Vitro Release of BR from the Developed NE

In order to determine the percentage of BR released from the developed NE formulations, the Agilent Fiber optics dissolution system (Agilent Technologies, Santa Clara, CA, USA) was utilized. Glass tubes were prepared by attaching from one side with cellophane membrane (Spectra/Por^®^ Membrane, MWCO: 12,000–14,000), Spectrum Laboratories Inc., Breda, The Netherlands) and then, 2 mL of each preparation was added to the tubes. The tubes were suspended in the device and immersed in 750 mL of PBS, pH 7.4, at 37 ± 0.5 °C, under a sink condition and allowed to rotate at 50 rpm. Spectrophotometric analysis was performed on the tested samples at designated intervals (0.25, 0.5, 1, 2, until 24 h) at λmax 264 nm [24]. Each experiment was performed in triplicate using the same batch.

### 3.6. Studying In Vitro Release of BR from the Developed NE Following Serum Incubation

The previous method was followed to evaluate the in vitro release study following the incubation of the formulations with an equal volume of 10% serum [64]. The purpose of in vitro release after serum incubation is to assess the behavior of the formulated naked or PEGylated NE in the biological setting, especially in the presence of serum proteins. All the measurements were performed in triplicate for the same batch, and the results have been expressed as mean ± standard deviation (SD).

### 3.7. Quantitative Determination of Serum Protein Adsorbed onto the Surface of NE

This study was performed in order to assess the amount of protein quantitatively adsorbed at the surface of the preparations. Accordingly, equal volumes of NE and fresh rat serum were incubated together at 37 °C for 30 min. The NE–serum mixture was then separated from bulk serum proteins using the previously mentioned method of running NE over a Sepharose CL-4B gel column [65]. Samples of NE formulations were collected in order to quantitatively assess the amount of protein associated on their surfaces by measuring the total lipid and total protein content. All the measurements were performed for the same batch in triplicate, and the results have been expressed as mean ± standard deviation (SD).

### 3.8. Hemolytic Activity

This experiment is a crucial signal in various biological studies. This test was performed to evaluate the tendency of the developed nanocarrier to rupture the red blood cells, releasing hemoglobin into the surrounding fluid. Blood was extracted from rats, utilizing a syringe containing drops of heparin, and the entire collected blood was spun at 1500× *g* for 10 min at 20 °C. After centrifugation, plasma was removed and substituted with an equal volume of PBS, pH 7.4, and then subjected to further centrifugation, three consecutive times. Erythrocytes were weighed and added to prepare 2% *w*/*v* solution in PBS, pH 7.4. Afterward, one mL of the NE formulation was incubated for 30 min with an equal volume of erythrocyte suspension in a water bath at 37 °C. Another centrifugation at 3000× *g* for 10 min was performed to separate the released hemoglobin from the erythrocytes. The supernatant was diluted with PBS 7.4 before measuring the absorbance at λmax 550 nm. Regarding the control, erythrocytes were incubated in PBS alone as a control, or they could be mixed with 1 mL of Triton-X 100 (1%) to achieve 100% hemoglobin release [10]. All the measurements were performed for the same batch in triplicate, and the results have been expressed as mean ± standard deviation (SD).

### 3.9. In Vitro Cytotoxicity Study

#### 3.9.1. Cell Line

MDA-MB-231 cancer cells were bought from the American Type Culture Collection (ATCC; Manassas, VA, USA) through the College of Science, King Faisal University, KSA. MDA cells were cultured in DMEM supplemented with 100 μg/mL of streptomycin, 100 U/mL of penicillin, 20 μg/mL of gentamicin, and 10% heat-inactivated FBS under 5% CO2/95% air at 37 °C.

#### 3.9.2. MTT Assay

This is a commonly performed in vitro cytotoxicity study used to evaluate cell proliferation and viability [66]. In the current investigation, the cytotoxicity of the naked and PEGylated BR-NE, as well as that of the BR solution, was examined against cancer cells, mainly MDA-MB-231 cells, using MTT assay. Using a 96-well plate, about 5000 cells were seeded and treated for 48 h with a certain concentration of free BR, BR-loaded NE, and blank NE. MTT dye was added to each well and incubated for 4 h. The media were detached, and each well was mixed with DMSO and shaken mechanically for 10 min. The optical density was then estimated at 570 nm [67].

### 3.10. Animals

#### 3.10.1. Declaration of Ethical Approval

All animal handling protocols in the current study were approved by and conducted in accordance with the Institutional Review Board (IRB) of King Faisal University, approval number KFU-REC-2022-SEP–ETHICS154. All the in vivo experiments were implemented in accordance with the guidelines of the Kingdom of Saudi Arabia and the research policies of IRB of King Faisal University, Hofuf, Saudi Arabia.

#### 3.10.2. Experimental Animals

The animals in the study were provided by the breeding center at King Faisal University, College of Science. Male Balb/c mice of about 8–10 weeks were used to prepare tumor-bearing mice models. Five million MDA tumor cells were subcutaneously inoculated into the right back of the animal to produce tumor-bearing mice. The animals were examined three times, weekly, at the injection site.

### 3.11. In Vivo Studies

#### Evaluating the Anti-Tumor Activity of BR-Loaded NEs

This study was performed in order to evaluate the anti-tumor influence of BR-loaded NE formulations on tumor-bearing mice. Following the inoculation of MDA tumor cells into the mice, tumor growth was monitored. Once the tumor volume reached around 200 mm^3^, the study was started. The animals were distributed among 6 groups, each of 8 mice, as follows:

Group 1: Treated with saline (control).

Group 2: Treated with BR solution (2 mg/kg).

Group 3: Treated with NE1 without BR (Blank 1)

Group 4: Treated with NE2 without BR (Blank 2)

Group 5: Treated with NE1 formulation (Naked NE)

Group 6: Treated with NE2 formulation (PEGylated NE)

All formulations were given to the animals via intravenous injection at a dose of 2 mg/kg through the tail vein. The volume of the tumor was estimated daily and calculated using the equation documented by Lee et al., where two measurements were performed using a caliper [68]:

Volume (mm^3^) = longer diameter × (shorter one) 2 × 0.52

The experiment was terminated upon the death of any mouse in either group [69].

### 3.12. Statistics

All values are presented as the mean ± SD of a minimum of three separate experiments. Student’s *t*-test was used to examine the statistical differences between the groups. Using SPSS statistical software, version 9 (IBM Corporation, Armonk, NY, USA), a one-way analysis of variance (ANOVA), followed by the post hoc test: Tukey’s Honestly Significant Difference test, was used to compare the data from the treatment groups with the data from the control group. Differences were considered statistically significant when *p* was <0.05.

## 4. Conclusions

The investigation successfully established that the targeted delivery of brucine and Nigella sativa oil via a PEGylated nanoemulsion system offered a prospective treatment approach for solid tumors. Surface modification drives the nanocarrier to enhance drug accumulation at the tumor site, which consequently improves the bioavailability, and suggests low systemic toxicity. The hemolytic assay proposed the biocompatibility of the formulation with red blood cells, supporting its safety for intravenous administration. The in vitro cytotoxicity assay exhibited lower IC50, suggesting higher anti-proliferative activity of the PEGylated nanoemulsion. Ultimately, the PEGylated BR-loaded nanoemulsion could significantly lower the tumor volume during the in vivo anti-tumor activity test. Overall, the study suggests potential synergism by providing an excellent nanocarrier combination between therapeutic agents and a natural bioactive compound. It emphasized the successful role of surface modification in prolonging the resident time of the nanocarrier in systemic circulation; however, comprehensive in vivo systemic toxicity studies are required to fully evaluate the safety profile of the formulation. Additionally, further studies on non-malignant cell lines are recommended to confirm the selectivity and safety of the formulation.

## Figures and Tables

**Table 1:** Characterization of developed BR-loaded NEs: Section 2.1.

Formulation	Particle Size (nm)	PDI	Viscosity (cp)	Zeta Potential (mV)	Drug Content %
NE1	515.9 ± 5.2	0.768	2.9 ± 0.30	−20.0 ± 1.15	98.6 ± 0.7
NE2	188.5 ± 3.3	0.211	3.4 ± 0.45	−1.61 ± 0.82	99.0 ± 1.0

**Table 2:** Constituents of the formulated BR-loaded NE formulations.

Constituents	BR (%*w*/*w*)	NS Oil (%*w*/*w*)	Cholesterol (%*w*/*w*)	Oleic Acid (%*w*/*w*)	PEG-DSPE (%*w*/*w*)	EPC (%*w*/*w*)	Dist. H_2_O to (%*w*/*w*)
NE1 (Naked NE)	0.5	15	0.4	0.3	0	0.12	100
NE2 (PEGylated NE)	0.5	15	0.4	0.3	0.5	0.12	100

## Data Availability

The original contributions presented in this study are included in the article. Further inquiries can be directed to the corresponding authors.
